# Enhancer Responses to Similarly Distributed Antagonistic Gradients in Development

**DOI:** 10.1371/journal.pcbi.0030084

**Published:** 2007-05-11

**Authors:** Robert P Zinzen, Dmitri Papatsenko

**Affiliations:** Department of Molecular and Cell Biology, Center for Integrative Genomics, University of California, Berkeley, California, United States of America; The Hebrew University, Israel

## Abstract

Formation of spatial gene expression patterns in development depends on transcriptional responses mediated by gene control regions, enhancers. Here, we explore possible responses of enhancers to overlapping gradients of antagonistic transcriptional regulators in the *Drosophila* embryo. Using quantitative models based on enhancer structure, we demonstrate how a pair of antagonistic transcription factor gradients with similar or even identical spatial distributions can lead to the formation of distinct gene expression domains along the embryo axes. The described mechanisms are sufficient to explain the formation of the anterior and the posterior *knirps* expression, the posterior *hunchback* expression domain, and the lateral stripes of *rhomboid* expression and of other ventral neurogenic ectodermal genes. The considered principles of interaction between antagonistic gradients at the enhancer level can also be applied to diverse developmental processes, such as domain specification in imaginal discs, or even eyespot pattern formation in the butterfly wing.

## Author Summary

The early development of the fruit fly embryo depends on an intricate but well-studied gene regulatory network. In fly eggs, maternally deposited gene products—morphogenes—form spatial concentration gradients. The graded distribution of the maternal morphogenes initiates a cascade of gene interactions leading to embryo development. Gradients of activators and repressors regulating common target genes may produce different outcomes depending on molecular mechanisms, mediating their function. Here, we describe quantitative mathematical models for the interplay between gradients of positive and negative transcriptional regulators—proteins, activating or repressing their target genes through binding the gene's regulatory DNA sequences. We predict possible spatial outcomes of the transcriptional antagonistic interactions in fly development and consider examples where the predicted cases may take place.

## Introduction

With the availability of complete genome sequences and quantitative gene expression data, it becomes possible to explore the relationships between sequence *features* of regulatory DNAs and the transcriptional responses of their associated genes [1–7]. Developmental genes regulated by multiple enhancer regions and their spatio–temporal dynamics of expression are of particular interest [8–11]. The enhancers of developmental genes, such as gap and pair-rule genes, interpret maternally deposited information and participate in the formation of progressively more complex expression patterns, thus increasing the overall spatial complexity of the embryo. In part, the information required to generate these downstream patterns (e.g., gap and pair-rule) is present in the enhancer sequences.

Much attention has been paid to the investigation of transcription factor binding motifs and motif combinations, and to interpreting their role in the formation of spatial gene expression patterns. [5,12,13]. However, some early enhancers of *Drosophila* contain virtually identical sets of binding motifs, yet they produce distinct expression patterns [6,14]. It has been argued extensively that binding site quality (affinity) and site arrangement within enhancers (grammar) contributes to the levels and precision of enhancer responses [6,15–21]. In fact, some experimental studies of differentially arranged binding sites confirm the dependence of enhancer response on distances between binding sites and on binding site orientation [6,16,22–24], and some structural enhancer features such as motif spacing preferences and characteristic binding site linkages. “Composite elements” and other syntactical features were identified in many model organisms using computational analyses of binding site distributions throughout entire genomes [5,25,26]. Recent studies involving in vivo selection of optimal binding-site combinations in yeast also revealed a number of preferred motif combinations and structural features [27]. Nevertheless, some phylogenetic studies indicate significant flexibility in the regulatory code [28–31].

The analysis of unrelated, structurally divergent, but functionally similar enhancers aids in defining the balance between the stringency of the functional *cis*-regulatory “code” and its flexibility as demonstrated by changes in primary enhancer sequence over the course of evolution. [6,18,32]. Requirements for multiple cofactors that influence transcription via protein–protein interaction complicate computational predictions and studies of enhancers. While known binding motifs are easy to find, most protein–protein interactions leave no clear *footprints* in the DNA sequence of enhancers—some developmental coregulators such as CtBP (C-terminal binding protein) and Groucho influence the transcriptional response through interactions with sequence-specific transcription factors (e.g., [33]). Finally, regulatory signals from enhancers must be transmitted to the basal transcriptional machinery; this involves enhancer–promoter communication of some sort, as well as the recruitment of mediator complexes [2,21,34–36]. Both aspects further complicate the in silico prediction and analysis of enhancer activity.

Until recently, most models explaining enhancer responses in development were largely qualitative [37,38]. Davidson's group [2,39] and Hwa's group [21] undertook quantitative modeling of enhancer–promoter interactions and investigated the responses of architecturally complex regulatory units. The elaborate nature of developmental enhancers in *Drosophila* was described in quantitative models introduced by Reinitz's group [1,7]. Here, we summarize some basic structural considerations and investigate mechanisms of enhancer regulation to demonstrate how such features may affect the transcriptional responses. Our quantitative analyses involve models based on the fractional occupancy of transcription factor binding sites present within enhancers [2,21,40,41]. On the one hand, the described models are similar to those developed by Hwa's group [21] as they consider structural enhancer details. On the other hand, the models include biological assumptions for developmental enhancers (i.e., quenching), similar to those introduced by Reinitz's group [7]. Technically, our models use a homotypic array (a unit containing a number of identical sites) of binding sites as an elementary unit for modeling.

Based on quantitative analysis of transcriptional responses, we analyze some models for developmental pattern formation. In particular, we explore the outcome of the interplay between two antagonistic transcription factors, an activator and a repressor. We demonstrate that a pair of antagonistic gradients with similar or even identical spatial distributions is sufficient to initiate stripes of expression of a downstream gene. Given that the antagonistic gradients may be deposited by the same localized or terminal signal (e.g., in the fly embryo) [42] or by a focal signal (e.g., in the case of a butterfly eyespot) [43], the models explain how initiation from a single point in space can lead to efficient gains in spatial complexity.

## Results/Discussion

### Successful Transcriptional States of Enhancers

The transcriptional state of enhancers of developmental genes is among major factors in developmental pattern formation [6–8,10]. If a transcription factor is present in a concentration gradient, the probability of that factor occupying a binding site in a target enhancer at a given position along the gradient depends on the factor's concentration at that position (coordinate). This logic suggests that in the case of activator and repressor gradients, calculating the probability of activator, but not repressor, binding (i.e., the successful transcriptional state resulting in transcription) may serve well to model the spatial expression patterns of the early developmental genes.

**Figure 1:**
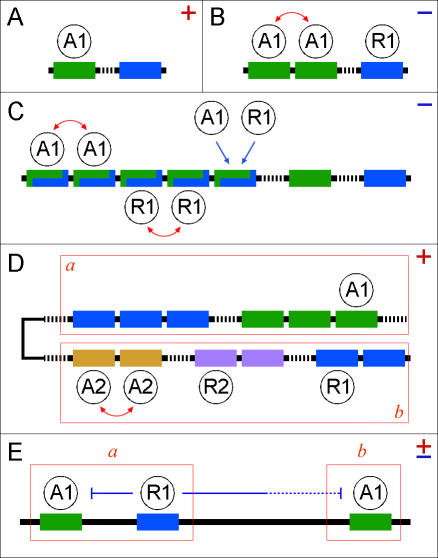
Known Elements of Enhancer Structure (A) A simple regulatory unit, containing an activator (green) and a repressor (blue) binding site. Binding of an activator, but not a repressor, is sufficient for transcriptional activation (indicated by “+”). (B) A cooperative array and a single repressor site. Red arrow shows the cooperative interaction between transcription factors. Repressor binding blocks enhancer activity (indicated by “−”). (C) A competitive array. Blue arrows show the competition between the activator and the repressor; red arrows show cooperativity. (D) A two-module enhancer (modules *a* and *b*) is able to respond to two groups of independent inputs. While the module *b* is repressed, the module *a* is active and the enhancer is active, as long as the short-range repression from module *a* does not reach module *b.* (E) A short-range repression can reach sites within the same module *a*, but fails to completely repress an activator site in the distant module *b*. The outcome of this interaction is a partial inactivation (± sign).

Let us consider an elementary enhancer, which contains two binding sites: one for an activator and one for a repressor. Let us assume that binding of the activator *A* in the absence of the repressor *R* brings the elementary two-site regulatory unit *i* (the enhancer; see Figure 1A) into a successful transcriptional state.

The equilibrium probability of the successful state *p_i_* depends on the binding probabilities of *A* (*p_A_*) and *R* (*p_R_*), which depend on the concentrations of the regulators ([*A*] and [*R*]) and on the binding constants (*K_A_* and *K_R_*) of the binding sites for the corresponding transcription factors (see Equations S1–S5 in Protocol S1):

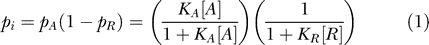
Extending this formula to multiple different activators or repressors may be easily obtained with the same logic (see Equation S6 in Protocol S1). Bintu and coworkers recently introduced a number of similar models, describing DNA–protein and protein–protein interactions on proximal promoters [21], where the authors used an “effective dissociation constant,” which is the inverse of the binding constant (*K*) used in this study.

### Cooperativity and Competition in Binding Site Arrays

Developmental enhancers usually contain homotypic or heterotypic binding site arrays for multiple activators and repressors [44]. The probability of achieving a successful transcriptional state for the binding site array (enhancer) *i*, containing *M* identical, noninteracting activator sites and *N* identical, noninteracting repressor sites, is equal to (see Equation S7 in Protocol S1):

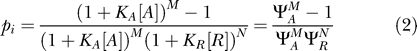
Here, Ψ is the sum of the statistical weights of molecular microstates for a homotypic site array and the denominator Ψ*_A_^M^*Ψ*_R_^N^* is the sum of the statistical weights for all microstates of the system (i.e., the partition function; see Protocol S1, “Binding site arrays”).

In such site arrays, bound transcription factors may cooperate or compete for binding. Let us consider a cooperative array as an element of enhancer architecture (Figure 1B). Assuming presence of lateral diffusion [41,45], equal binding affinities for all sites in the array and expressing cooperativity *C* as the ratio between the second and the first binding constants, one can approximate the sum of statistical weights Ψ of all possible molecular microstates for a cooperative array as follows (see Equations S8 and S9 in Protocol S1):

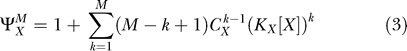



Binding sites for an activator and a repressor may overlap, and the corresponding proteins compete for binding. Well-known examples in *Drosophila* development include Bicoid and Krüppel [46], Caudal and Hunchback [44], and Twist and Snail [6]. The classic example outside *Drosophila* is the competition between CI and Cro in the phage *lambda* switch [47]. The sum of microstates for a competitive site array, containing *M* overlapping *A/R* binding sites (Figure 1C; also see Figure S1 and Equations S8–S12 in Protocol S1), can be approximated by:

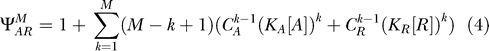



In addition to competitive interactions, this model also includes homotypic cooperative interactions between the regulators (see Equations S10–S12 in Protocol S1).

### Independent Modules, Distances, and Short-Range Repression

Structural elements within an enhancer (single sites or entire site arrays) may be distributed over extended genomic regions (thousands of bases, e.g., the Drosophila sna enhancer) [48,49]. In these cases, the distant regulatory elements within the enhancer may represent relatively independent units—modules [15,26] (see Figure 1D). Each independent module may include a single binding site or a binding site array. Redundancy of the enhancer elements (binding sites and modules) is a well-known biological phenomenon [44]. If the modules within an enhancer are independent from one another, bringing any one module into a successful transcriptional state may be sufficient for bringing the entire enhancer into a successful state, even if another module(s) is repressed.

Given the probabilities *p_i_* of successful states of all *i* independent modules or enhancers (Equations 1–4), the probability *P_Enc_* of the multimodule enhancer being in a successful state is equal to:

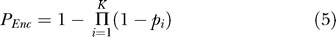
This is the reverse probability of the enhancer being in an inactive state, which is the product of the probabilities of each independent module being in an inactive state (1 − *p_i_*); Reinitz's group [1,7] implemented similar expressions for the quenching mechanism. While distinct modules may provide simultaneous responses to different inputs, multiple equivalent modules may allow for the boosting of an enhancer's overall response to a single input [50] (see Figure S1E and S1F).

In practice, however, the modules may not be completely independent from each other. Short-range repression and other factors (discussed below) may be involved in distance-dependent module responses [22–24,48]. Let us consider an enhancer containing two modules, *a* and *b*. Module *a* contains an activator site and a repressor site; module *b* contains an activator site only (see Figure 1E). Potentially successful enhancer states include all combinations in which at least one activator molecule is bound. However, the mixed state *K^a^_A_*[*A*]*K^a^_R_*[*R*] is always inactive as the repressor, and the activator sites in the module *a* are “close”. If module *b* is not “*too* far” from module *a,* short-range repression from *a* may reach the activator site in *b*. We can account for this possibility (and for its extent) by introducing a multiplier *δ,* depending on distance between the modules *a* and *b* (see also Equations S14–S16 in Protocol S1):

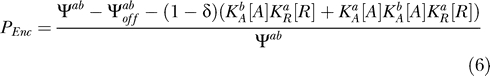
In this formula, Ψ*^ab^* is the sum of weights for all microstates, and Ψ*^ab^_off_* is the sum of weights for the microstates that are always inactive (see Protocol S1, Equation S14). If modules *a* and *b* are “far,” *δ =* 1; if they are “close,” *δ =* 0. If the distance between *a* and *b* is somewhere in between, so that a repressor bound in *a* partially affects the activator bound in *b*, we could introduce a distance function *δ = f*(*x*) (0 ≤ *δ* ≤ 1), where *δ* depends on the distance *x* between *a* and *b* (and perhaps other variables, such as the repressor type). However, all we currently know about the distance function is that short-range repression is effective at distances less than 150–200 bases, and long-range repression may spread through entire gene loci (i.e., 10–15 kb [23,24,48]). Without exact knowledge about the distance function, the module concept (Equation 5) allows modeling of distance-dependent responses, but only in a binary close/far (yes/no) fashion.

### Embryo Axis Patterning by a Pair of Antagonistic Regulators

Most of the enhancer response models (Equations 1–6) consider inputs from two antagonistic gradients, but enhancers may be under the control of a larger number of regulators (see Figure 1D). However, gradients of some of these regulators may either have similar spatial distributions (e.g., Dorsal and Twist) [51], or non-overlapping spatial expression domains (e.g., Krüppel and Giant) [37]. Therefore, in many cases the combination of all inputs may be parsed down to one or more pairs of antagonistic interactions. Based on the described quantitative models approximating enhancer responses (see above), we analyzed possible spatial solutions produced by gradients of two antagonistic regulators.

**Figure 2:**
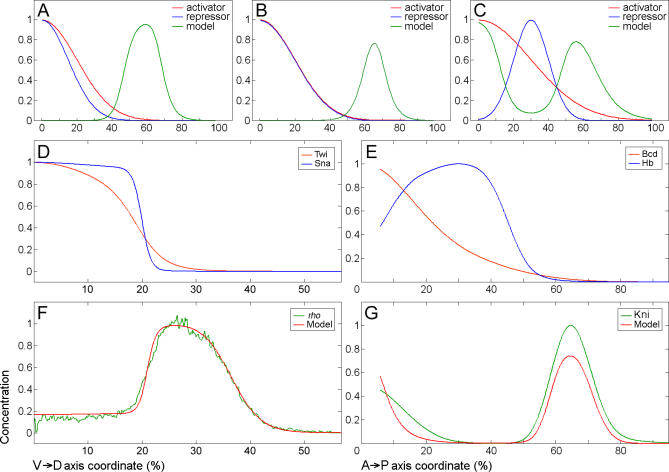
Outcomes of Two Antagonistic Gradients (A–C) Possible spatial distributions of activator (red), repressor (blue), and the corresponding predicted responses (green). Input gradients here were approximated as Gaussian functions (see other functions in Figure S2). (A) The activator has a slightly wider expression domain. (B) The activator and the repressor have identical distributions. (C) The peak of the repressor gradient is shifted relatively to the peak of the activator; expression of the downstream gene is “split” into two domains. (D) Spatial patterns of Twist (red) and Snail (blue). (E) Spatial patterns of Bicoid (red) and Hunchback (blue). (F) Spatial pattern of *rho* (green) and the *rho* model (in red). (G) Spatial pattern of Knirps (green) and a *knirps* model (red).

The examples in Figure 2A–2C demonstrate that the spectrum of possible enhancer responses is quite rich. One surprising result of these simulations is that even *identically* distributed antagonistic gradients can yield distinct spatial expression patterns such as stripes (Figure 2B). We identified conditions for the “stripe” solutions using differential analysis of the site occupancy function shown in Equation 1. For example, if both regulators are distributed as identical gradients and if their concentrations and binding constants are equal (*K_A_* = *K_R_*; [*A*] = [*R*]), then it is sufficient to identify conditions for the maximum of a site-occupancy function *y*(*x*) depending on the spatial coordinate *x*:

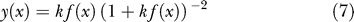
In this variant of Equation 1, *k* is the product of absolute concentration of the regulators [*Abs*] and the binding constant *K_A_* (*k* = *K_A_*[*Abs*]). The function *f*(*x*) is the distribution of the relative concentration (0 ≤ *f*(*x*) ≤ 1) of the transcription factors along the spatial coordinate *x* (i.e., the embryo axis). The function's maximum *y*′(*x*) = 0; x > 0 is *f* (*x*) = 1/*k*. In the Gaussian, logistic, and exponential decay forms of the function *f*(*x*) (see details in [52]), the maximum 1/*k* exists only if *K_A_*[*Abs*] > 1 (i.e., if binding constants and/or the absolute concentrations are high) (see also Figure S2). In the simple case (Equation 7), the absolute value of the fractional occupancy at the maximum is not very high (0.25); adding more sites or modules (see Figure S1) allows for the function's values to approach 1 (see Figure 2B).

However, if the antagonistic gradients are not identical (e.g., if the activator gradient is “wider” than the repressor gradient), the solutions for the stripe expression are more robust (Figure 2A). Shifting the peak of the activator gradient relative to the repressor gradient produces even more robust stripe patterns, as in the case of classical qualitative models [37], where a repressor “splits” or “carves out” the expression of a target gene (Figure 2C).

The formation of distinct gene expression domains (e.g., stripes) in response to similarly or even identically distributed gradients is of interest because this mechanism can lead to the very efficient gain of spatial complexity in just a single step: based on primary sequence, enhancers of target genes can translate two similarly distributed gradients into distinct gene expression domains or stripes. Such similarly distributed antagonistic gradients may come about by induction due to a single maternal gradient or due to a terminal (focal) signal emanating from a discrete point or embryo pole. The general pattern formation mechanism in the case described can be represented as follows: (1) maternal/terminal signal initiates two antagonistic gradients; and (2) interactions between the two gradients produce multiple stripe patterns. In an extreme case (e.g., Figure 2B), the described “antagonistic” mechanism could use only a single gradient/polar signal to produce multiple stripes of target gene expression.

The interaction between two antagonistic gradients is an example of a feed-forward loop. Due to a cascade organization of the developmental transcriptional networks, feed-forward loops are among the most common network elements (network motifs); a detailed analysis of the feed-forward networks and potential solutions can be found in a recent work by Ishihara et al [53].

### Requirements for *rhomboid* and *knirps* Expression

To explore the interplay of antagonistic gradients in detail, we considered particular examples, such as the regulation of *rhomboid (rho)* by gradients of Twist and Snail and the regulation of *knirps* by the maternal gradients of Hunchback and Bicoid [54].

The enhancer associated with *rho* directs localized expression in ventral regions of the neurogenic ectoderm (vNEs) [51]. The *rho* vNE enhancer, as well as enhancers of other vNE genes such as *ventral nervous system defective (vnd),* is activated by the combination of Dorsal and Twist, but is repressed by Snail in the ventral mesoderm [13,51]. Both Twist and Snail are targets of the nuclear Dorsal gradient, which is established by the graded activation of the Toll receptor in response to maternal determinants [55]. The Twist and Snail expression patterns occupy presumptive mesodermal domains in the embryo, yielding slightly distinct protein distributions. Our recent quantitative analysis indicates that the boundaries of *rho* and *vnd* expression are defined largely by the interplay of the two antagonistic Twist and Snail gradients (see Figure 2D and 2F) [6], and the expression patterns of *rho* and *vnd* resemble the predicted solutions shown in Figure 2A. The patterning mechanism in this case can be represented as follows: (1) a terminal signal (Toll/Dorsal gradient) initiates two similar antagonistic gradients, Twi and Sna; and (2) Twi and Sna gradients produce multiple (distinct) stripe patterns (*rho*, other vNE genes).

Another example of the interplay between an activator and a repressor gradient is the early expression of the gap gene *knirps* in response to maternal gradients of Bicoid and Hunchback. Bicoid and Hunchback are deposited maternally and have similar, but distinct distributions—high in the anterior and low in more posterior regions of the embryo (see Figure 2E). The graded drop-off of the *knirps* repressor Hunchback at 50%–60% egg length is steeper than that of the *knirps* activator Bicoid. This is similar to the theoretical case shown in Figure 2C, where a narrow repressor “splits” a wider activator expression domain, thus producing two peaks of expression of the downstream gene. Known enhancer elements of *knirps* drive *kni* expression in the anterior and the posterior embryo domains and contain binding sites for Bicoid, Hunchback, Caudal, Tailless, and Giant [44,56–58]. However, *tailless, caudal,* and *giant* are downstream of Bicoid; it is likely that these and some other genes participate in the later maintenance of *kni* expression. It has been extensively argued that gap genes (and *hunchback*) stabilize their patterns along the anterior–posterior axis by mechanism of mutual repression [49]. At later stages (after cycle 14), the inputs from Bicoid and Hunchback into *knirps* regulation may stabilize fluctuations in *knirps* expression and fluctuations in the entire gap gene network due to mutual repression. Dynamic models from Reinitz's group based on slightly different logistic response functions support the sufficiency of Bicoid and Hunchback in the establishment of the early *knirps* expression [59].

To explore the role of Bicoid and Hunchback interplay in the early expression of *knirps,* quantitative expression data for Bicoid, Hunchback, and Knirps were downloaded from the FlyEx database [60], and models simulating the *knirps* enhancer response were generated based on Equations 1–4. One model assumed that Bicoid and Hunchback bind independently from each other; another model assumed that there is an interference (possibly competition) between the Bicoid and the Hunchback sites (Equation 7: competitive binding). Fitting the available quantitative data with the models (see parameter values in Table 1) shows that both models are sufficient to explain the posterior expression of *knirps.* However, the competitive model (Figure 2G) also predicts the anterior expression of *knirps.* This result was especially striking, as the anterior *knirps* expression data were not included in some of the fitting tests. Bicoid and Hunchback motifs are quite different, so it is unlikely that this is a case of direct competition for overlapping binding sites. Other mechanisms may account for the negative interaction between the two regulators; for instance, binding of Bicoid may prevent Hunchback dimerization [61] and/or efficient binding.

**Table 1:**
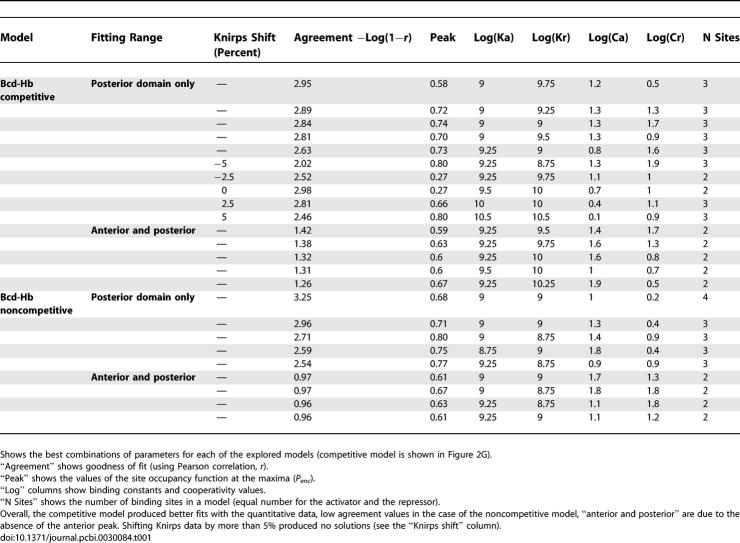
Successful Parameter Combinations for *knirps*

Shifting the *knirps* expression data by more than 5% along the anterior–posterior axis (see Materials and Methods) results in reduction of the data-to-model fit quality for the posterior *kni* expression domain (see Table 1 for exact parameter values). The robustness of *knirps* regulation was emphasized earlier [59,62], and the present analysis using site occupancy confirms that the interplay of the two antagonistic gradients, Bicoid and Hunchback, is sufficient to explain the initial formation of both the anterior and the posterior strips of *knirps* expression.

### Response of Mutagenized *rho* Enhancers In Vivo

To test the models describing gene response to antagonistic gradients, we introduced mutations in the *rho* enhancer and compared the expression patterns produced by the reporter gene in vivo with the simulated expression patterns simulated in silico (Equations 1–6). Specifically, the models for *rho* and *vnd* expression predicted the following [6]: (1) The position of the dorsal expression border of *rho* is highly sensitive to Twist and/or its cooperativity with Dorsal. Reducing Dl–Twi cooperativity or Twist–Twist cooperativity shifts the dorsal border ventrally. (2) The number of independent elements (groups of closely spaced Dorsal-Twist-Snail sites, or “DTS” elements) contributes to the expression pattern of *rho* and *vnd* according to Equation 5 (boost): a higher number of DTS elements in *vnd* is responsible for the shift of the ventral *vnd* expression border relatively to *rho* [6].

**Figure 3:**
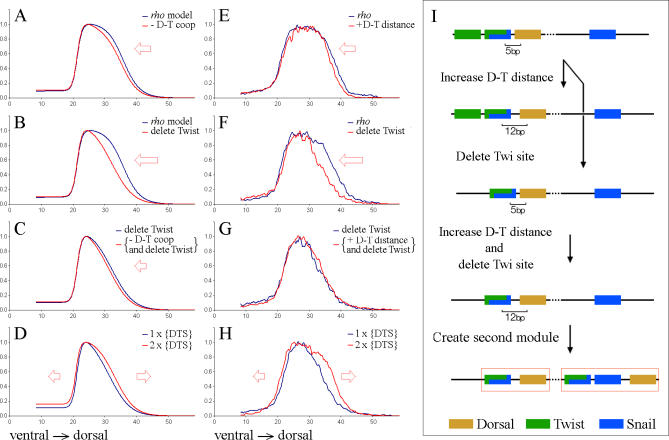
In Vivo Versus In Silico Responses of a Modified Enhancer Predicted responses in silico (A–D) and the observed responses in vivo as quantified in transgenic flies (E–H) of mutagenized *rho* enhancers. (I) Mutations incorporated in the enhancer structure (see Materials and Methods for sequences). Reducing Dorsal-Twist cooperativity in silico or increasing the distance between the Dorsal and the Twist sites in vivo results in a slight ventral shift of the dorsal expression border (compare (A) with (E)). Deleting a weak Twist site shifts the dorsal border ventrally (B,F). The combined effect of both mutations is similar to the deletion of Twist (C,G). Creation of a second DTS element (red boxes) results in an expected overall “boost” in expression, thereby shifting both the ventral and dorsal expression boundaries, “widening” the *rho* stripe (D,H).

These two specific predictions, based on the model analysis and simulations, were tested by modifying the structure of the minimal *rho* enhancer. First, the distance between the Dorsal and the Twist sites in the DTS element was increased (see Figure 3). The increased distance between the two sites reduced the cooperative potential between the Dorsal and Twist sites. Indeed, the observed effect in vivo is consistent with the effect of the same mutation simulated in silico, causing a ventral shift of the dorsal border of the reporter gene expression (compare Figure 3E with 3A). An additional mutation eliminating the weaker Twist site from the DTS element affects Twist–Twist cooperativity in the enhancer and shifts the dorsal *rho–lacZ* expression border. In fact, the combined effect produced by these two mutations in vivo (Figure 3G; compare with 3C) and the deletion of the weak Twist site alone (Figure 3F; compare with 3B) demonstrate shifts of the dorsal expression border of the *rho-lacZ* transgene in concordance with the models. Last, a second DTS module was introduced into the *rho* enhancer in the context of the previous two mutations. The predicted in silico effect is a “boost” in expression, resulting in the shift of both ventral and dorsal expression borders. Again, the predicted changes in the expression pattern were observed in vivo—not only were the positions of the ventral and the dorsal border shifted (Figure 3H; compare with 3D), but the overall level of expression of this transgenic construct appears higher (unpublished data).

The described in vivo tests of the in silico predictions using site-directed mutagenesis of the *rho* enhancer have demonstrated that though the quantitative models based on fractional site occupancy are approximations, they can produce reasonable predictions for the response of complex regulatory units (such as fly enhancers) to gradients of transcriptional regulators.

### The Interplay of Two Gradients May Explain a Broad Spectrum of Developmental Patterns

Using transcriptional response models and quantitative expression data, we demonstrated how two similar terminal gradients can determine stripes of expression of downstream genes. Related examples are quite frequent in development. For instance, the posterior stripe of *hunchback* is the result of activation by Tailless and repression by Huckebein [63,64]. As in the case with Twist and Snail, the posterior gradient of Tailless is slightly broader than the gradient of Huckebein. Therefore, the mechanisms of posterior *hunchback* expression may be similar to the mechanisms shown in Figure 2A, 2B, 2D, and 2F. However, while the examples above involve direct transcriptional regulation in the embryonic syncytial blastoderm, extracellular morphogen gradients may produce similar outcomes if the cellular response is transcriptional in nature.

**Figure 4:**
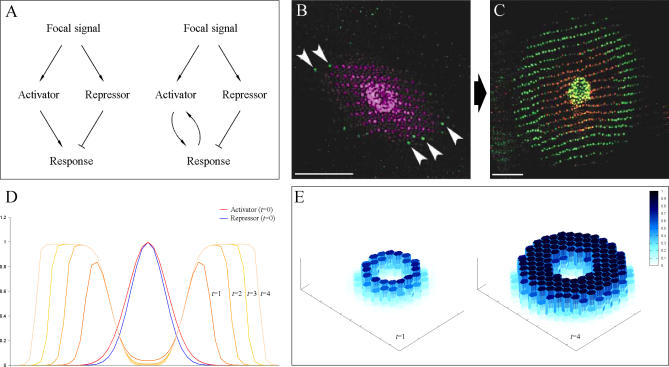
Possible Extensions of the Antagonistic Model (A) Feed-forward network motif (left) and the motif with a feedback to the activator (right). (B,C) Expansion of the *engrailed* expression pattern in the eyespot of the developing butterfly wing (image adapted from C. Brunetti [67]: *engrailed* expression, green; *spalt* expression, purple and red). White arrowheads signify initial *engrailed* pattern. (D) A dynamic model for the network motif with feedback reproduces the outward expansion of the *engrailed* ring in the eyespot. The initial response of *engrailed* is equivalent to that shown in Figure 2A; consequently. the *engrailed* extends itself “outward” from the focus. (E) 2.5-Dimensional plot of (D) for time points *t* = 1 and *t* = 4.

Formation of eyespot patterns in butterfly wings is an elegant example of axial (here focal) patterning in a cellular environment (see Figure 4A). The interplay between Notch and Distalless specifies the position of focal spots and intervein midline patterns in the butterfly wing [65]. Subsequent Hedgehog signaling from the focal spots is believed to induce the formation of concentric rings of gene expression and the pigmentation of the eyespots in the adult butterfly wing [66]. Known targets of the Hedgehog gradient are the butterfly homologs of *engrailed* and *spalt* [67]. Initially, both genes are expressed around the focal spot, but at later stages an external ring of *engrailed* expression appears around the *spalt* expression pattern (see Figure 4B and 4C). In the case of *engrailed* pattern formation, a simplified mechanism [67] may include elements of the following feed-forward network: (1) focal signal (focal spot/Hedgehog signaling) initiates two antagonistic gradients, the activator Engrailed and the repressor Spalt; and (2) subsequent interactions between Engrailed and Spalt produce multiple ring patterns. An extension of the model in Equation 1, (*k* is the rate of synthesis and *c* is the rate of decay; *d*[*R*]/*dt* = 0) reproduces the dynamic changes in the *engrailed* pattern (Figure 4A, 4D–4E):

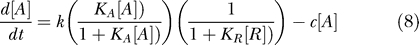
Examples of axial or focal patterning using a single source of signaling or a combination of similar antagonistic gradients are common. The interplay between maternal *hunchback* and maternal *nanos* during development of the short germ-band insect *Schistocerca* is an example of axial patterning similar to the interplay between Bicoid and Hunchback [68]. Specification of segments during insect limb development is comparable to the mechanisms of Twist/Snail interplay and the butterfly eyespot formation [69]. Nature uses many combinations of signals and gradients in pattern formation, but the most effective mechanism/combination may be one that allows maximal informational gain in a minimal number of steps. From this perspective, the interplay between similar or identical gradients is of significant interest.

## Materials and Methods

### Quantitative expression data.

Quantitative distribution data for Dorsal, Twist, and Snail were published previously [6]. Quantitative expression data for mRNA levels of mutated *rho* enhancers were generated by in situ hybridization (the data are available at the DV*Ex* database: http://www.dvex.org). Multiplex in situ hybridization probes were used for colocalization studies, including co-stainings for the endogenous mRNAs and *lacZ* reporter gene expression as described previously, and confocal microscopy and image acquisition were performed as described [6]. In short, signal intensity profiles of sum projections along the dorso–ventral axis of mid-nuclear cleavage cycle of 14 embryos were acquired using the ImageJ analysis tool (National Institutes of Health, http://rsb.info.nih.gov/ij). Background signals were approximated by parabolic functions and subtracted according to existing methods [70]. Online programs for the automated background subtraction and data alignment are available from the University of California Berkeley Web resource (http://webfiles.berkeley.edu/∼dap5). After background subtraction, the data were resampled and aligned according to the position of Snail gradient and the distribution of endogenous *rho* message. Expression datasets for anterior–posterior genes were downloaded from the FlyEx database (with options: integrated, without background) [60]. In all cases, signal amplitude was normalized to the 0–1 range, and the data was resampled to 1,000 datapoints along the coordinate of the corresponding axis. In all models, we used the relative concentration multiplied by a maximal absolute concentration. This absolute concentration is an independent unknown parameter (range, 10^−8^–10^−9^ *M*) equal for all reaction components.

### Mutagenesis of *rho* enhancers.

The minimal *rho* enhancer [6] was mutated via site-directed mutagenesis in pGem T-Easy (Promega; http://www.promega.com) using the following primers: Dl-Twi distance, RZ65mut: 5′-GTTGAGCACATGTTTACCCCGATTGGGGAAATTCCCGG-3′; deletion of Twist site, RZ66mut: 5′-GGCACTCGCATAGATTGAGCACATG-3′; creation of a second DTS, RZ67mut: 5′-GCAACTTGCGGAAGGGAAATCCCGCTGCAACAAAAAG-3′; and RZ68mut: 5′-CACACATCGCGACACATGTGGCGCAACTTGC-3′.

Mutated enhancers were cloned into the insulated P-element injection vector E2G as described previously [13]: constructs were introduced into the D. melanogaster germline by microinjection as described previously [71]. Between three and six independent transgenic lines were obtained and tested for each construct; results were consistent across lines.

### Fitting models to data and exploration of parameter space.

To fit our models with actual quantitative data, we maximized the agreement *r* (Pearson association coefficient) between the model output predictions and the observed (measured) expression patterns:

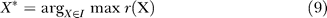
The best set of parameters X^*^ from the parameter space *I* is defined by the binding constants, cooperativity values, and the number of binding sites. We used a standard hill-climbing algorithm (full neighborhood search) for the main parameter space (e.g., [72]). For each identified maximum, we measured the value of the site occupancy function and discarded maxima that produce site saturation values below selected thresholds, as well as such that are located beyond selected realistic parameter ranges for binding constants and cooperativity values. All maxima producing the highest data-to-model agreement were found multiple times, suggesting that exhaustive mapping of the parameter space was achieved. Fitting “shifted data” (wrong data) for Knirps was performed by exploring exactly the same parameter space and exactly the same number of seed points for each shift value. Quantitative gene expression data for dorso–ventral genes are available at http://www.dvex.org; the analysis tool “E-response,” fitting utilities, and online data-treatment programs are available at the University of California Berkeley Web resource http://webfiles.berkeley.edu/∼dap5.

## Supporting Information

Figure S1 Dependence of Enhancer Response on Models, Structural Elements, and Mechanisms Panels on the left, (A,C,E,G,I), show transcriptional responses; panels on the right (B,D,F,H,J) show ratios between the response functions.
(A) Disagreement between two models for a cooperative array (Equations S8 and S9).
(B) Ratio between the two models for different numbers of sites, *M*.
(C) Disagreement between models for cooperating competitive arrays (blue, Equation S10; green, Equation S11; red, Equation S12).
(D) Ratio between model Equation S11 and model Equation S12 for different values of *M*.
(E) Responses of one-, two-, and three-module configurations for a six-site cooperating competitive array.
(F) Ratios between model Equation S11 to model Equation S10 (green) and Equation S12 to Equation S10 (red).
(G) Response of two modules next to each other (in blue), at a distance of 80 bases and at infinite distance (in green).
(H) Ratio between models for separated and neighboring modules. Numbers show the distance between the modules in bases; the distance dependence (*δ*) is an arbitrary sigmoid function with saturation at ∼150 bases.
(I) Dependence of enhancer response on molecular mechanism of repression.
(J) Ratios for direct competition (in green), short-range repression (in red), and the long-range repression (in purple). Repressor concentration is constant in all cases.
(543 KB TIF)

Figure S2 Dependence of Response on the Shape of Identical Gradients (A–D) Similar antagonistic gradients may have different shapes, which correspond to different form of the function (*f*(*x*) in Equation 7. However, the presence of the response function maximum (*y*(*x*) in Equation 7 is independent from the form of the function *f*(*x*). All simulations were performed with the same concentration values and the same binding constant values.
(543 KB TIF)

Protocol S1 Detailed Model Description (156 KB DOC)

## Abbreviations

DTS: Dorsal-Twist-Snail
*rho,*: *rhomboid;*
*vnd,*: *ventral nervous system defective;*
vNE: ventral region of the neurogenic ectoderm
