# Supplementary material for: Enhancer Responses to Similarly Distributed Antagonistic Gradients in Development
Source: PLoS Comput Biol. 2007 May 11;3(5):e84. doi: 10.1371/journal.pcbi.0030084 (PMC1866357; doi:10.1371/journal.pcbi.0030084)
Supplement: Supplementary file 1 [file pcbi.0030084.sd001.doc]

### Supplement

**Enhancer Responses to Similarly Distributed Antagonistic Gradients in Development**

Robert P. Zinzen and Dmitri A. Papatsenko

Department of Molecular and Cell Biology

Center for Integrative Genomics

University of California, Berkeley

CA 94720-3204

Fractional site occupancy

Consider a binding of an activator (regulator) protein *A* to its binding site *S*:

$[A]+[S]\leftrightarrow[AS]$ (1S)

Association equilibrium constant *K_A_* of this reaction is equal to:

$K_{A}=\frac{[AS]}{[A][S]}$ (2S)

Here, we are interested in the ratio between the bound *S* - [*AS*] and the total amount of *S* -[*S*]+[*AS*] or probability *p* of the activator binding:

$p=\frac{[AS]}{[S]+[AS]}$ (3S)

One can use eq (2S) to substitute [*AS*] using association constant:

$p=\frac{K_{A}[A][S]}{[S]+K_{A}[A][S]}=\frac{K_{A}[A]}{1+K_{A}[A]}$ (4S)

Successful transcriptional states of an enhancer

Let’s assume, that binding of the activator *A*, but not the repressor *R* brings an elementary 2-site regulatory unit *u* (an enhancer) into a successful transcriptional state (see **Figure 1 A**). The probability of the successful state *p_u_* can be obtained from eq (4S):

$p_{u}=p_{A}\left(1-p_{R}\right)=\frac{K_{A}[A]}{(1+K_{A}[A])(1+K_{R}[R])}$ (5S)

Often, several repressors are involved into the regulation of developmental and other genes; in addition, there are cases, when more than one activator is required. In the absence of cooperative interactions between the bound transcription factors, probability *p_u_* can be computed using eq. (5S) or its extensions for the case of multiple regulators.

For, instance, both Dorsal and Twist are required to activate *rhomboid* (*rho*), while Snail serves as the *rho* repressor [1, 2]. For a hypothetical 3-site (Dorsal+Twist+Snail) *rhomboid* enhancer, the eq. (5S) takes the form:

$p_{rho}=p_{Dl}p_{Twi}\left(1-p_{Sna}\right)=\frac{K_{Dl}[Dl]K_{Twi}[Twi]}{(1+K_{Dl}[Dl])(1+K_{Twi}[Twi])(1+K_{Sna}[Sna])}$ (6S)

Binding site arrays

Formula 5S (or 6S) can be extended to the case with multiple binding sites. To calculate the binding probability for multiple sites, one has to consider all possible combinations of occupied and empty states for all binding sites. For instance, for 2 activator binding sites all possible combinations will include 0-0, 0-1, 1-0, 1-1. This corresponds to the sum $\Psi$of weights for the microstates: $\Psi_{A}^{2}=1+2K_{A}[A]+(K_{A}[A])^{2}$. $\Psi$is a partition function or the sum of statistical weights for all microstates available to the system. For a microstate with *n* molecules bound, its unnormalized statistical weight is the product of the number of possible arrangements (Binomial coefficients, in our case), concentration term, $[A]^{n}$ and a binding energy term $K_{A}^{n}$, ($K_{A}=e^{-\Delta E/RT}$). Any of the “occupied” states will have the same outcome – activation, so the sum of the successful, “active” combinations will be equal to (1+K_a_[A])^2^-1. For enhancer *u,* containing *M* identical, non-interacting activator sites, and *N* identical non-interacting repressor sites, the probability of successful transcriptional state is equal to:

$p_{u}=\frac{(1+K_{A}[A])^{M}-1}{(1+K_{A}[A])^{M}(1+K_{R}[R])^{N}}=\frac{\Psi_{A}^{M}-1}{\Psi_{A}^{M}\Psi_{R}^{N}}$ (7S)

For short, we designate here and below the sum of all possible molecular microstates of a homotypic site array (1+*K_A_*[*A*])*^M^* as $\Psi_{A}^{M}$.

Assuming identical binding properties for all sites in the cooperative array and expressing cooperativity *C* as the ratio between the second and the first binding constants, one can approximate Ψ for a cooperating array of *M* activator binding sites as follows:

$\Psi_{X}^{M}=1+\sum_{k=1}^{M} \frac{M!}{k!(M-k)!}C_{A}^{k-1}(K_{X}[X])^{k}=1+\frac{\left(1+C_{X}K_{X}[X]\right)^{M}-1}{C_{X}}$ (8S)

The binomial form of the equation is due to the binary nature of transcription factor binding (bound/unbound). It is also possible to use another approximation for a cooperative array, where only “all-neighboring” molecular states of are taken into account:

$\Psi_{X}^{M}=1+\sum_{k=1}^{M} \left(M-k+1\right)C_{X}^{k-1}(K_{X}[X])^{k}$ (9S)

For a sequence containing 4 “closely spaced” binding sites (4-site array, *M*=4), the “all-neighboring” states will include the following: [1-1-0-0], [0-1-1-0], [0-0-1-1] (*k*=2, 1-bound, 0-empty). It is easy to see that the total number of the “all-neighboring” states is equal to M-k+1. In the presence of lateral diffusion and high cooperativity (*C*>1), non-neighboring states would undergo regrouping into the all-neighboring states, so the eq. 9S should be a reasonable approximation for a cooperating array (see **Figure 1S A, B**).

When the binding sites for an activator and a repressor overlap, the corresponding proteins can compete for binding. Let us consider an array of competing binding sites– another known structural element of enhancer architecture (see **Figure 1 C**). Enhancer response mediated by a competitive site array containing *M* overlapping *A/R* binding sites can be described using the following polynomial expression:

$\Psi_{AR}^{M}=\left(1+K_{A}[A]+K_{R}[R]\right)^{M}=1+\sum_{k=1}^{M} \sum_{j=0}^{k} \frac{M!}{(M-k)!(k-j)!j!}(K_{A}[A])^{k-j}(K_{R}[R])^{j}$ (10S)

Besides competing for the sites in the array, the activator and the repressor molecules can also be involved in cooperative interactions, *A*-*A* and *R*-*R*. Introducing cooperativity *C_A_* for the activator and *C_R_* for the repressor into the polynomial sequence yields a response model for a competitive array with cooperative interactions as follows:

$\begin{matrix} \Psi_{AR}^{M}=1 + \sum_{k=1}^{M} \frac{M!}{k!(M-k)!}\left(C_{A}^{k-1}(K_{A}[A])^{k}+C_{R}^{k-1}(K_{R}[R])^{k}\right) + \\ \sum_{k=2}^{M} \sum_{j=1}^{k-1} \frac{M!}{(M-k)!(k-j)!j!}C_{A}^{k-j-1}(K_{A}[A])^{k-j}C_{R}^{j-1}(K_{R}[R])^{j} \end{matrix}$ (11S)

The second term in the equation 11S accounts for the “exclusive” states of the array, in which *either* activator *or* repressor molecules are bound. The last term in the equation accounts for the “mixed” states, when some sites are bound by the activator and some by the repressor. While the “exclusive” states can be modeled like cooperative arrays, it is not quite clear how cooperative interactions behave in the “mixed” states. Activator and repressor molecules may prevent each other from binding and/or may affect lateral diffusion. In either case, the relative contribution of the mixed states to the sum of weights is small. Therefore, a simplest practical approximation for a competitive array with cooperative interactions is the same “all-neighboring” state model:

$\Psi_{AR}^{M}=1+\sum_{k=1}^{M} \left(M-k+1\right)\left(C_{A}^{k-1}(K_{A}[A])^{k}+C_{R}^{k-1}(K_{R}[R])^{k}\right)$ (12S)

Distances between binding sites and short-range repression

Binding sites may form closely spaced arrays or may be distributed over substantial distances in genome. In the latter case, the binding sites (or even entire binding site arrays) may work independently: repression form one site (or an array of sites) never reaches the others. Response of an enhancer containing *i* independent site arrays may be approximated as follows:

$P_{Enc}=1-\prod_{i=1}^{K} (1-p_{i})$ (13S)

However, short-range repression and other factors may be involved in distance-dependent responses. Let us consider an enhancer containing two modules, *a* and *b*. Module *a* contains an activator site with the binding constant $K_{A}^{a}$ and a repressor site with the binding constant$K_{R}^{a}$; module *b* contains an activator site only, with the binding constant $K_{A}^{b}$ (see **Figure 1 E**). All molecular microstates of the entire enhancer are described by the following combination of occupied/empty states: $\Psi^{ab}=\left(1+K_{A}^{a}[A]\right)\left(1+K_{A}^{b}[A]\right)\left(1+K_{R}^{a}[R]\right)$. Potentially successful enhancer states include all combinations in which at least one activator molecule is bound. If the module *b* is “far” from *a*, i.e. short-range repression from the repressor in *a* cannot reach *b,* then the binding of an activator molecule to its site in *b* brings the entire enhancer into an active state, even if the repressor is bound to *a*. In that case, only 3 states are always inactive: the empty state (one), the state, when only repressor is bound to *a*, $K_{R}^{a}[R]$ and the mixed state$K_{A}^{a}[A]K_{R}^{a}[R]$:

$P_{Enc}=\frac{\Psi^{ab}-\left(1+K_{R}^{a}[R]+K_{A}^{a}[A]K_{R}^{a}[R]\right)}{\Psi^{ab}}=\frac{\Psi^{ab}-\Psi_{off}^{ab}}{\Psi^{ab}}$ (14S)

In equation 14S, $\Psi_{off}^{ab}$ is the sum of the inactive states. Equation 14S can also be obtained by considering the independent modules *a* and *b* using formula 13S:

$P_{Enc}=1-\left(1-\frac{K_{A}^{a}[A]}{\left(1+K_{A}^{a}[A]\right)\left(1+K_{R}^{a}[R]\right)}\right)\left(1-\frac{K_{A}^{b}[A]}{1+K_{A}^{b}[A]}\right)$ (15S)

Equations 14S and 15S are equivalent. However, if the module *b* is not “*too* far” from the module *a*, short-range repression from *a* may reach the activator site in *b*. We can account for this possibility (and for its extent) by introducing a multiplier *δ*, depending on the distance between the modules *a* and *b*:

$P_{Enc}=\frac{\Psi^{ab}-\Psi_{off}^{ab}-\left(1-\delta\right)\left(K_{A}^{b}[A]K_{R}^{a}[R]+K_{A}^{a}[A]K_{A}^{b}[A]K_{R}^{a}[R]\right)}{\Psi^{ab}}$ (16S)

If modules *a* and *b* are “far”, *δ =*1, if they are “close”, *δ =*0. If the distance between *a* and *b* is somewhere in-between, so that a repressor bound in a partially affects the activator bound in *b*, we could introduce a distance function *δ =f*(*x*) $\left(0\leq\delta\leq1\right)$, where *δ* depends on the distance *x* between *a* and *b* (and perhaps other variables, such as the repressor type). For instance, consider a competitive array of 6 binding sites separated into 2 independent modules (see **Figure 1 F**). Let $\Psi_{A}^{3}$ and $\Psi_{A}^{6}$be the sum of states for 3 and 6 cooperating activator sites (equation 9S) and let $\Psi_{AR}^{3}$and $\Psi_{AR}^{6}$ be the sum for the total number of states for these competitive arrays (equation 12S). Then, the response of an enhancer containing two modules, each of three sites (in competitive arrays) at a distance *x*_0_ (*δ*_0_ *=f*(*x*_0_)) will be equal to:

$P_{Enc}=p_{(1x6)}+\delta_{0}[p_{(2x3)}-p_{(1x6)}]=\frac{\Psi_{A}^{6}-1}{\Psi_{AR}^{6}}+\delta_{0}\left[1-\left(1-\frac{\Psi_{A}^{3}-1}{\Psi_{AR}^{3}}\right)^{2}-\frac{\Psi_{A}^{6}-1}{\Psi_{AR}^{6}}\right]$ (17S)

The two-module (2x3) configuration results in additional successful or partially successful states as compared to the one-module (1x6) enhancer configuration (compare responses in **Figure 1S G, H**); i.e. states where a repressor bound to one module is not silencing the other module completely. The partially successful states can be accounted for with the help of the distance function *δ*.

Mechanisms of repression and influence of genomic sequences

One of our initial assumptions was that the presence of a bound repressor molecule prevents transcriptional activation (see 5S). However, the example of distance dependency (equations 14S to 17S) demonstrates exceptions to this rule related to a particular molecular mechanism – short-range repression. Clearly, exact enhancer responses also depend on the specific molecular mechanisms and interactions. Experimental studies of repression have revealed several possible mechanisms of repression: (*i*) direct competition, (*ii*) short or long-range repression (quenching), and (*iii*) competitive inhibition (squelching) [3-5]. Direct competition between activators and repressors for binding sites may or may not be coupled with short-range (or long-range) repression – the case of short-range repression was considered above in detail, but if there is only physical competition, the response would be different. All “mixed” states, i.e. states when both activator and repressor molecules are present will be successful (compare with equations 14S-17S for short-range repression). Substituting the polynomial expression for a competitive site array into the basic formula (equation 5S) and subtracting states in which the activator is not present yields a model for direct competition alone:

$p=\frac{(1+K_{A}[A]+K_{R}[R])^{M}-(1+K_{R}[R])^{M}}{(1+K_{A}[A]+K_{R}[R])^{M}}$ (18S)

For *M*=1 the mixed states are not available, in which case there is no difference between direct competition and direct competition coupled with short-range repression.

In the case of long-range repression, mixed states (eq. 16S) are not successful, as the repressor bound anywhere in the locus blocks transcription. Long-range repression makes binding site arrangements less sensitive to distances between activator and repressor sites. For the same competitive array of 6 sites split into 2 independent modules (see equation 17S), the enhancer is active if an activator is bound to either module and the second module is not repressed (it may be empty):

$P_{Enc}=1-\left[1-\left(\frac{\Psi_{3A}-1}{\Psi_{3}}\right)\left(\frac{\Psi_{3A}}{\Psi_{3}}\right)\right]^{2}$ (19S)

**Figure 1S I, J** compares responses of the same enhancer structure (2x3 array), but considering different mechanisms of repression.

Yet another possible mechanism of repression, competitive inhibition or squelching, demonstrates a possible effect of interactions outside DNA sequences on enhancer response. Repressors may bind transcriptional activators in solution, thus preventing them from binding to the target sites in regulatory units. In these cases, the transcriptional activator *A* may be bound either to the repressor in solution [*AR*], or to binding sites in enhancers [*AE*]. As [*AE*] can be assumed to be small, a close approximation for the effective activator concentration is the sum of free activator plus activator bound by the repressor: [*A*]=[*A_free_*]+[*AR*]. Substitution of [*AR*] with binding constants for the repressor-activator reaction gives the amount of activator in solution available for binding to DNA:

$[A_{free}]=[A]/(1+K_{R}[R])$ (20S)

The adjusted concentration of the activator can be applied to a model including other elements of enhancer structure. For an array of *M* activator sites (exclude the repressor terms from equation 5S), the competitive inhibition is equivalent to direct competition (equation 12S) with the exception that *K_R_* in the former case is the constant for the repressor-activator binding in solution.

The presence of non-functional (background) binding sites in the genome would have an effect similar to direct or competitive inhibition. Berg, Von-Hippel, and others [6, 7] suggested that up to 90% of the amount of a transcription factor is involved in non-specific binding, so the concentration of regulators in all models may be adjusted according to equation 20S, where *K_R_*[*R*] is the term representing genomic DNA. In many cases, the constant reflecting the effect of the genome *g*=(1+*K_R_*[*R*]) may be ignored however, as the *absolute* concentrations of the regulators are typically not known.

Figure legends

**Figure 1S. Dependence of enhancer response on models, structural elements, and mechanisms**

Panels on the left (*A*, *C*, *E*, *G*, *I*) show transcriptional responses, panels on the right (*B*, *D*, *F*, *H*, *J*) show ratios between the response functions. (*A*) Disagreement between two models for a cooperative array (equations 8S and 9S). (*B*) Ratio between the two models for different numbers of sites, *M*. (*C*) Disagreement between models for cooperating competitive arrays (blue, eq. 10S; green, eq. 11S; red, eq. 12S). (*D*) Ratio between model eq. 11S and model eq. 12S for different values of *M*. (*E*) Responses of one, two and three module configurations for a 6-site cooperating competitive array. (*F*) Ratio between the model eq. 11S to model eq. 10S (green) and eq 12S to eq 10S (red). (*G*) Response of two modules next to each other (in blue), at a distance 80 bases and at infinite distance (in green). (*H*) Ratio between models for separated and neighboring modules. Numbers show the distance between the modules in bases, the distance dependence (*δ*) is an arbitrary sigmoid function with saturation at ~ 150 bases. (*I*) Dependence of enhancer response on molecular mechanism of repression. (*J*) Ratios for direct competition (in green), short-range repression (in red) and the long-range repression (in purple). Repressor concentration is constant in all cases.

**Figure 2S. Dependence of response on the shape of identical gradients**

(*A*-*D*) Similar antagonistic gradients may have different shape, which corresponds to different form of the function (*f*(*x*) in eq. 7). However, the presence of the response function maximum (*y*(*x*) in eq. 7) is independent from the form of the function *f*(*x*). All simulations were performed with the same concentration values and the same binding constant values.

References

1.	Stathopoulos A, Levine M: **Whole-genome analysis of Drosophila gastrulation**. *Curr Opin Genet Dev.* 2004, **14**:477-484.

2.	Zinzen RP, Senger K, Levine M, Papatsenko D: **Computational models for neurogenic gene expression in the Drosophila embryo**. *Curr Biol.* 2006, **16**:1358-1365. Epub 2006 Jun 1356.

3.	Levine M, Manley JL: **Transcriptional repression of eukaryotic promoters**. *Cell.* 1989, **59**:405-408.

4.	Cai HN, Arnosti DN, Levine M: **Long-range repression in the Drosophila embryo**. *Proc Natl Acad Sci U S A.* 1996, **93**:9309-9314.

5.	Barolo S, Levine M: **hairy mediates dominant repression in the Drosophila embryo**. *Embo J.* 1997, **16**:2883-2891.

6.	von Hippel PH, Berg OG: **Facilitated target location in biological systems**. *J Biol Chem.* 1989, **264**:675-678.

7.	Bolouri H, Davidson EH: **Transcriptional regulatory cascades in development: initial rates, not steady state, determine network kinetics**. *Proc Natl Acad Sci U S A.* 2003, **100**:9371-9376. Epub 2003 Jul 9325.
